# Phase transitions in cortical dynamics explain improved information processing under attention

**DOI:** 10.1186/1471-2202-14-S1-P126

**Published:** 2013-07-08

**Authors:** Nergis Tomen, Udo Ernst

**Affiliations:** 1 Institute for Theoretical Physics, University of Bremen, 28359 Bremen, Germany

## 

A major aspect of cortical information processing is its rapid adaptation to the actual behavioral demands. Highly representative for such processes is selective visual attention, which has been shown to improve performance in visual perception (see e.g. [1]). The neural mechanisms supporting these effects have been investigated in electrophysiological studies: Besides firing rate modulations and tuning curve changes in visual cortical neurons, they revealed a strong increase of oscillations in the gamma frequency band (35-90 Hz) [2]. These findings indicate that gamma oscillations might play an important role in optimizing information processing under attention, but their functional role is currently not yet understood.

The dynamics of cortical networks exhibit certain properties that have been suggested to be linked to optimization of information processing: criticality and balance. Experimental studies indicate that cortical networks operate near a 'critical' state in which scale-free avalanches of spike events occur [3], generating neural patterns which are 'rich' in structure [4]. These critical states are indicators of a phase transition from an asynchronous regime of activity in the network towards a synchronous regime. Furthermore, it has been observed that excitatory and inhibitory synaptic currents to cortical neurons are balanced [5], which allows cells to detect rapid changes in the stimulus and provides an explanation for the high variability of neural activity.

Here we explore the relationship between the phase transition towards synchrony, balance, and enhanced stimulus representation in a simple network of integrate-and-fire neurons driven by an external stimulus. By increasing the efficacy of recurrent couplings, attention improves spontaneous synchronization and renders activation patterns for different stimuli more distinct. We compare this result to experimental evidence demonstrating that object representation in cortical local field potentials (LFPs) is improved in macaque monkeys under attention [6], and find a near-perfect match between model and LFP data.

By analyzing the LFPs and avalanche statistics of the simulated network activity, we show that the enhancement of discriminability of LFP power spectra is strongly correlated with phase transitions towards synchrony. More importantly, our results indicate that networks operating near phase transitions reside in a regime where a dramatic enhancement of stimulus representation may require only fine modulations of synaptic strengths. Taken together, our study implies that attention drives the cortex towards a critical state, inducing oscillatory activity and hereby improving the discriminability of different stimuli represented in the corresponding network activity. Furthermore, our framework suggests a novel role for synchronization in cortical information processing.
